# Gonorrhœa

**Published:** 1881-04-20

**Authors:** A. F. Durham

**Affiliations:** Georgia


### GONORRHŒA.

BY A. F. DURHAM, M. D., OF GEORGIA.

Without going into an elaborate discussion or description of this seemingly familiar disease, I will just briefly state the symptoms that usually mark its ingress and progress in the acute form:

The subject on rising, generally from the fourth to the seventh morning after innoculation, if a male, will notice a slight itching at the mouth of the urethra, which will be found slightly glued together, an unusual redness, and on squeezing the glans the discharge of a very small quantity of a perfectly transparent or white opaque fluid, and on urinating, a little tingling or stinging sensation. If the disease is not arrested at this point, the above symptoms will be followed in a few hours by a decided burning on urinating, extending from half an inch to the whole length of the canal, with increased discharge, thickened, a decided yellow color, and, if the attack is very acute, merging into a greenish cast.

In the course of thirty-six or forty-eight hours at farthest, *the vexatious* and distressing symptom of cordee proclaims the stride of the disease, causing the patient not unfrequently, late at night especially, to utter not very pious ejaculations, exclamations, rash threats and harsh promises, the while invoking the embrace of Morpheus.

I will here, rather digressively, state that urethral chancre is sometimes mistaken for this disease, but a peculiar lancinating pain at a certain spot, continual but aggravated by pressure, at each discharge of urine will render the differential diagnosis pretty clear, and if unarrested the secondary symptoms of syphilis, as bubo, sore-throat and a characteristic cutaneous eruption will put all doubt at rest.

*Treatment*—Close observation, with an experience of thirty years, has convinced me that of all the thousand and one "boasted and infallible" remedies, there is but one specific treatment, and that is the abortive.

A solution of argenti nitratis 60 grains to the ounce (no less) will, —or has in my hands at least—invariably destroyed the specific character of the disease, leaving nothing but a simple inflammation to deal with. But this should be resorted to only in the incipiency, or when the disease has not extended above an inch up the urethra, and not left to an ignorant or untaught patient. If it is, it will likely prove unsatisfactory,—to say the least—if not dangerous. The proper way to apply it is, to secure the urethra about half an inch behind the diseased portion with the thumb and index finger of one hand, while the syringe is carefully introduced with the free hand. The solution should be thrown in with considerable force to insure contact with the whole diseased surface. Just previous to this the canal should be washed out with soap-suds or simple water as warm as can be conveniently borne. Immediately follow the nit. arg. with a syringeful of a solution of chloride of sodium.

The following used every three, four or six hours will generally subdue the inflammation within forty-eight hours:

℞ Zinci acetatis,<br>

Plumbi acetatis,<br>

Morphæ acetatis,........................................ aa grs. iv.<br>

Pulv. accacia,........................................ ʒ ij.<br>

Aq. rosæ, vel camphoræ,........................................ f ℥ viij

M. Sig. A syringeful immediately after urinating.

Bitartrate of potassa and sulphate of magnesia in equal portions should be taken in teaspoonful doses every two or three hours until the bowels become soluble.

As it seldom happens that a case is seen in time to avert it, I briefly give the treatment I usually pursue, which, in a vast majority of cases, is entirely successful. I direct the patient to put a heaping tablespoonful of equal parts of bitart. potass. and magnesia sulphatis into a quart of water and take a wine-glassful of the solution every three or four hours until the bowels become lax, and then continue *pro re nata.* Meantime use the following:

℞ Potass. permanganatis,........................................ grs. iv.<br>

Zinci sulphatis,........................................ grs. xxiv.<br>

Morphiæ sulphatis,........................................ grs. iv.<br>

Acid gallic,........................................ grs. iv.<br>

Acaciæ,........................................ ʒ ij.<br>

Aq. camph. vel. rosæ........................................ f ℥ viij.

To get the full benefit of the medicine, the patient should be directed to make sufficient pressure on the perineum to prevent ingress into the bladder, by sitting on a chair or stool, placing a ball or some convenient body on the perineum and resting on it.

The urethra should be first fully distended with air by pumping into it with the syringe, securing the end of the penis between the thumb and index finger while the instrument is being withdrawn. The distention should be kept up a minute at least, and the urethra pressed from the scrotum forward with one fore-finger, which will press all the pus out of the lacunæ. This manipulation should be followed by urinating, and this immediately by the medicated fluid, which should be retained two or three minutes in the manner above described, and contact of the whole diseased surface is thus secured.

This should be repeated every four hours for two or three days, when the discharge ceases and the disease is cured.

I always use anodynes and diuretics internally, though it may not always be necessary.

The following is a favorite of mine, and seldom fails to accomplish all that could be desired: Ether nitrosi, acid nitros, tr. gelseminum, tr. opii camphorata and aq. menthæ vel cinnamon, mixed in proper proportions and taken in one or two teaspoonful doses *ter die*, half an hour after meals, diluted with sweetened water.

The cordee is easily controlled by small enemata of cold water per rectum during the day, and a mixture of camphor and hyoscyami suspended in flax seed or slippery elm infusion at night. When the disease becomes chronic I add either, balsam copaiba, or fir to the above internal mixture, or give in solid form, or when the patient is debilitated and anemic combine them with iron.

If the case is very obstinate I use chloride of zinc, one to three grains to the ounce, with the same proportion of acetate or muriate of morphia. When it is practicable, in the chronic form, I insert a metallic bougie into the bladder at bed-time, allowing it to remain all night and follow it with the injection in the morning as soon as the patient has voided urine, repeating once or twice during the day. The most obstinate cases readily yield to this treatment in a few days.

The diet in the acute form should be rather bland and unstimulating. In the chronic, where the iron is indicated, I allow a generous diet, with porter, ale or a drink of gin or fine corn whisky two or three times a day.

One word more. When the disease has lost its specific character occasional coition is beneficial, in two ways at least: it benefits the relaxed urethra and has a tendency to prevent and cure stricture.

